# Influence of Body Composition on Post-Exercise Parasympathetic Reactivation of Firefighter Recruits

**DOI:** 10.3390/ijerph18010339

**Published:** 2021-01-05

**Authors:** David J. Cornell, Sabrina E. Noel, Xiyuan Zhang, Kyle T. Ebersole

**Affiliations:** 1 Health Assessment Laboratory, University of Massachusetts Lowell, Lowell, MA 01854, USA; 2 Center for Population Health, University of Massachusetts Lowell, Lowell, MA 01854, USA; xiyuan_zhang@uml.edu; 3 Department of Physical Therapy and Kinesiology, University of Massachusetts Lowell, Lowell, MA 01854, USA; 4 Department of Biomedical and Nutritional Sciences, University of Massachusetts Lowell, Lowell, MA 01854, USA; sabrina_noel@uml.edu; 5 Human Performance and Sport Physiology Laboratory, University of Wisconsin-Milwaukee, Milwaukee, WI 53211, USA; 6 Department of Occupational Sciences and Technology, University of Wisconsin-Milwaukee, Milwaukee, WI 53211, USA; ebersole@uwm.edu

**Keywords:** autonomic nervous system, heart rate recovery, obesity, body mass index, waist circumference, percent body fat, tactical athletes

## Abstract

Firefighters have a sustained risk for experiencing a sudden cardiac event after completing a fire call. Heart rate recovery (HRR) can be utilized to characterize autonomic nervous system (ANS) recovery and has been linked to cardiac events. Research suggests that body composition influences post-exercise HRR responses in non-firefighter populations. The purpose of this study was to examine the influence of body mass index (BMI), waist circumference (WC), and percent body fat (BF) on the HRR response of firefighter recruits. BMI (kg·m^−2^), WC (cm), and BF (%) data from 57 firefighter recruits were collected. HRR (b·min^−1^) data were collected at completion (HR_0_), as well as 15 (HR_15_), 30 (HR_30_), 45 (HR_45_), 60 (HR_60_), 120 (HR_120_), and 180 (HR_180_) seconds following a submaximal step test, and commonly utilized clinical HRR indices were calculated (ΔHRR_30_, ΔHRR_60_, ΔHRR_120_, and ΔHRR_180_). After controlling for sex, linear mixed regression models did not identify significant interactions between body composition (*p*s > 0.05) and HRR response across time. However, significant (*p*s < 0.05) indirect semi-partial correlations were identified between BF and ΔHRR_30_ (*r*_sp_ = −0.31) and ΔHRR_60_ (*r*_sp_ = −0.27), respectively. Reducing overall BF (vs. BMI or WC) should be prioritized to improve the post-exercise ANS recovery of firefighter recruits.

## 1. Introduction

Sudden cardiac deaths (SCDs) account for roughly half of the line-of-duty deaths among United States (U.S.) firefighters [1], and for every SCD, there are an estimated 17–25 additional non-fatal line-of-duty cardiovascular events (stroke, heart attack, etc.) [2]. While many of these SCDs occur during fire-suppression activities, or shortly after suppression activities [3], the odds of a firefighter experiencing a SCD after a fire call remain 2.2 to 10.5 times higher than during nonemergency duties [4]. Based on epidemiologic evidence, it has been suggested that an incomplete physiological recovery of firefighters after a fire call may be placing these individuals at risk for experiencing SCD or other cardiovascular events, and that this incomplete physiological recovery could be due to improper regulation of the autonomic nervous system (ANS) [5,6].

Heart rate recovery (HRR), or the reduction in heart rate (HR) after exercise, has been previously associated with all-cause mortality and risk of cardiovascular events [7]. Since the early phase of HRR reflects the cardiac vagal reactivation of the parasympathetic nervous system (PSNS) of the ANS [8], it has been suggested that a greater HRR may produce an “antiarrhythmic effect” due to enhanced PSNS control [9]. Due to the high HR response achieved during fire-suppression activity [10,11], it has been hypothesized that improperly elevated sympathetic nervous system (SNS) activation after fire-suppression activities may be contributing to this elevated risk of SCD [5]. Therefore, understanding influencers of HRR may be of particular importance to the firefighter population.

Previous research indicates that obesity level is associated with a blunted post-exercise HRR response in non-firefighter populations [12,13,14,15,16]. Coupled with clinical research that has identified reductions in PSNS activity among obese individuals [17,18,19], including as a result of weight gain [20], this blunted HRR response could be due to a lack of PSNS re-engagement after exercise. Given the high prevalence of obesity among both active-duty firefighters [21] and recruits [22,23], and the considerable SNS activity associated with firefighting [5], understanding the influence of body composition on the HRR response of firefighters, and identifying which measures of body composition are most associated with the HRR response of firefighters, is needed.

Therefore, the purpose of the current study was to cross-sectionally examine the influence of body composition on the overall HRR response, as well as discrete windows of PSNS reactivity characterized by clinical measures of HRR, among firefighter recruits. It was hypothesized that measures of central adiposity and body fat, compared to overall obesity-level, would be most associated with measures of HRR.

## 2. Materials and Methods

### 2.1. Participants

Participants were recruited from Midwest U.S. firefighter recruit training academies associated with urban fire departments. Participants were considered eligible to participate if they were older than 18 years of age and were cleared for full participation within their training academy. Eligible participants were then included if they (1) were not taking any prescribed medication for a symptomatic illness; (2) did not sustain an injury or have surgery on their knees, hips, or ankles in the last year; (3) were not previously diagnosed with a heart condition or experienced chest pain or dizziness during exercise; (4) were not currently pregnant; and/or (5) had not been instructed by a physician to refrain from participating in exercise or physical activity.

Based on these criteria, all recruited participants were eligible to participate and a convenience sample of 57 firefighter recruits (51 men, 6 women) volunteered to participate in this study (Table 1). This study was conducted in accordance with the Declaration of Helsinki, and all study protocols were approved by the Institutional Review Board at the University of Wisconsin-Milwaukee (Protocol Number: 13.180), with all participants providing written informed consent.

### 2.2. Procedures

All data were collected cross-sectionally in group testing format at the training academy facility associated with the fire department of the firefighter recruits. Data were specifically collected indoors and at room temperature in a designated exercise space for the training academy. All participants were wearing standard athletic clothing (i.e., t-shirt, athletic shorts, and athletic shoes), and data were collected in the morning (between the hours of 1000 and 1100). All recruited participants agreed to participate in all data collection procedures, and no participants were lost due to drop-out.

#### 2.2.1. Body Composition Data

Body composition of each participant was characterized via body mass index (BMI), waist circumference (WC), and percent body fat (BF). All body composition data were collected according to standardized testing methods developed by the American College of Sports Medicine (ACSM) [24] and endorsed by both the International Association of Fire Fighters (IAFF) and International Association of Fire Chiefs (IAFC) [25]. To ensure consistency, the same research staff member collected body mass, height, WC, and skinfold measure data across all participants.

In brief, body mass (kg) and height data (cm) were collected while each participant was barefoot and wearing athletic clothing. Data were collected using a mechanical beam scale (Health-o-Meter Professional, Pelstar LLC, McCook, IL, USA) and were measured to the nearest 0.1 kg and 0.5 cm, respectively. Based on these collected body mass and height data, BMI (kg·m^−2^) was then calculated for each participant (BMI = body mass/height^2^) [26]. The WC (cm) of each participant was measured at the height of their iliac crest using a cloth gulick tape measure (Creative Health Care Products, Inc., Ann Arbor, MI, USA) and recorded to the nearest 0.1 cm [26].

The BF of each participant was estimated using the Jackson and Pollock three-site skinfold method [27]. Specifically, skinfold measures (mm) of each participant were collected using a Lange skinfold caliper (Beta Technology, Santa Cruz, CA, USA) and were measured to the nearest 1.0 mm. Based on the Jackson and Pollock three-site skinfold prediction equations [27], skinfold measures were taken from the right triceps, pectoral, and subscapular locations of men, and the right triceps, abdominal, and suprailiac locations of women [24]. Using these skinfold measures, the BF (%) of each participant was then estimated using the corresponding equation [27] and Siri equation [28].

#### 2.2.2. Heart Rate Data

HRR was characterized after completing a submaximal step test routinely utilized within both firefighter [22,29] and non-firefighter populations [30], which requires participants to step up and down on a 40 cm box to the beat of a metronome set to 90 b·min^−1^ for 5 min [31]. Upon conclusion, participants sat quietly on the box for a total of 3 min. HR (b·min^−1^) data were subsequently collected at completion (HR_0_), as well as 15 s (HR_15_), 30 s (HR_30_), 45 s (HR_45_), 60 s (HR_60_), 120 s (HR_120_), and 180 s (HR_180_) post-test recovery period. All heart rate data were collected using Polar T31i monitors (Polar Electro, Lake Success, NY). The PSNS reactivation of each participant was characterized by calculating the change in HR 30 s (ΔHRR_30_ = HR_0_−HR_30_), 60 s (ΔHRR_60_ = HR_0_−HR_60_), 120 s (ΔHRR_120_ = HR_0_−HR_120_), and 180 s (ΔHRR_180_ = HR_0_−HR_180_) post exercise [8].

### 2.3. Statistical Analyses

Three separate linear mixed models with random effects and repeated measure procedures were utilized to examine the influence of body composition variables (BMI, WC, and BF) on HRR response across time (HR_0_, HR_15_, HR_30_, HR_45_, HR_60_, HR_120_, and HR_180_). Semi-partial correlations determined the association between body composition variables (BMI, WC, and BF) and PSNS reactivation via commonly utilized clinical ΔHRR indices (ΔHRR_30_, ΔHRR_60_, ΔHRR_120_, and ΔHRR_180_) [6]. Sex (men/women) was controlled for as a covariate in all statistical analyses, which were conducted using SAS version 9.4 software (SAS Institute, Cary, NC, USA), and an alpha of 0.05 determined statistical significance.

## 3. Results

Descriptive data regarding the HRR responses and ΔHRR indices of participants are displayed as mean (SD) in Table 2. No significant interactions between HRR and BMI (β = 0.01 ± 0.06, *F*_1,339_ = 0.04, *p* = 0.840), WC (β = 0.02 ± 0.02, *F*_1,339_ = 0.52, *p* = 0.470), or BF (β = −0.01 ± 0.05, *F*_1,339_ = 0.01, *p* = 0.913) were identified. However, significant indirect semi-partial correlations were identified between BF and ΔHRR_30_ (*r*_sp_ = −0.31, *p* = 0.010) and ΔHRR_60_ (*r*_sp_ = −0.27, *p* = 0.023), respectively. All other semi-partial correlations were not statistically significant (*p*s > 0.05) (Table 3).

## 4. Discussion

The purpose of this study was to examine the influence of body composition on the post-submaximal exercise HRR response, as well as discrete windows of PSNS reactivity, of firefighter recruits. It was hypothesized that measures of WC and BF would influence the HRR and PSNS reactivity responses to the greatest extent. Contrary to this hypothesis, results of this study indicate that no measure of body composition influenced the overall HRR response of firefighter recruits across the 180-s window of recovery. However, BF was significantly associated with ΔHRR_30_ and ΔHRR_60_, which are considered to be indices of PSNS reactivity and are commonly utilized in a clinical context [8].

The fact that body composition did not influence the overall HRR response of firefighter recruits across the overall recovery window in the current study differs from previous research demonstrating significant relationships between a variety of body composition metrics and HRR responses [12,13,14,15,16]. However, the majority of previous studies did not examine HRR responses across an entire 180-s window of recovery and only utilized ΔHRR_60_ and/or ΔHRR_120_ metrics to characterize the HRR response of participants. That said, although the current study incorporated the inclusion of additional ΔHRR metrics, only the “fast phase” of the HRR response was examined [8]. Since this initial “fast phase” of the HRR response largely represents the PSNS reactivation of the ANS [8], the potential influence of body composition on the SNS withdrawal of the ANS (i.e., the “slow phase” of the HRR response) remains unknown. Due to recent research also demonstrating a lack of ANS recovery 10 min post submaximal exercise among active-duty firefighters [6], future research should utilize newer techniques of HRR assessment that collect continuous HR data across a longer window of time [8], in order to further elucidate the potential influences of body composition on overall ANS recovery.

Similar to previous research, the current study also examined commonly utilized clinical ΔHRR indices, but in contrast, only BF was significantly associated with the ΔHRR_30_ and ΔHRR_60_ indices. While the results of this study are consistent with previous research demonstrating relationships between ΔHRR_60_ and BF [12], no significant associations between ΔHRR_60_ and BMI were observed. These results differ from research conducted within non-firefighter populations that have identified significant associations between ΔHRR_60_ and BMI [12,15]. It is possible that BMI may not appropriately capture the influence of body composition on PSNS reactivation among firefighter recruits due to limitations associated with BMI as a measure (i.e., a lack of consideration of muscle mass) [32]. In addition, although it has been suggested that central adiposity may cause dominance of the SNS over the PSNS [33], the current work did not identify a significant relationship between ΔHRR_60_ and WC, which differs from previous research that has identified significant relationships between ΔHRR_60_ and WC in non-firefighter populations [12,14].

Taken together, results of the current study suggest that body composition may influence the ANS recovery of firefighter recruits differently than the general U.S. population. Due to the role that firefighters play in public safety, and the extreme physiological demand placed on these individuals as a result of their occupation-related duties [5], these differences between firefighter recruits and other general U.S. populations are important considerations for practitioners working within the firefighting sector. However, the large range in BMI (21.0–38.1 b·min^−1^) and WC (77.0–105.7 cm) reported in the literature [12,13,14] complicates the ability to compare firefighter populations to other populations. As a result, it is possible that certain levels of BMI and/or WC (i.e., “cut-points”) may result in a significant influence on HRR responses, and further investigations with larger sample sizes are warranted within the firefighter population.

Nevertheless, results of the current study indicate that overall body fat (i.e., BF) and not surrogate measures of obesity and central adiposity (i.e., BMI and WC, respectively), may be more implicated in the mechanisms associated with obesity-related ANS dysregulation noted in the literature [17,18,19]. Therefore, practitioners should examine measures of BF (vs. BMI or WC) when assessing post-fire call cardiac event risk among firefighters. Since an estimated 33–52% of career firefighters in the U.S. are classified as obese [21,34,35], practitioners should prioritize the reduction of overall BF, and not necessarily measures of BMI or WC, when trying to improve the PSNS reactivation of firefighter recruits in preparation for active-duty work. However, although previous research has identified improvements in PSNS activity as a result of weight loss [20], the most efficacious interventions to create such adaptations within the firefighter population are currently unknown. Future research should examine the influence of a variety of health and fitness interventions (e.g., aerobic exercise, resistance exercise, dietary behavior change, etc.) on BF adaptations among firefighters.

### Strengths and Limitations

Although the results of the current study provide unique and valuable data describing the influence of body composition on HRR responses of underrepresented population in the scientific literature (i.e., firefighter recruits), the power of the study is limited due to the small sample size (*n* = 57) and the use of skinfold measures to estimate BF, which have a standard error of 3.6–3.9% compared to criterion methods of BF assessment [36]. In addition, all participants were part of Midwest U.S. firefighter training academies, and were not yet active-duty firefighters, limiting the generalizability of the results. There were also far fewer female firefighter recruits than male firefighter recruits (6 vs. 51, respectively), making it difficult to examine any potential concomitant mediation of sex on the influence body composition on HRR responses. However, it should be noted that 10.5% of the sample population in the current study were women, which is more than the percentage of active-duty firefighters currently in the U.S (4.7%) [37]. Finally, the HRR response was only characterized after submaximal exercise in the current study. Since previous research suggests different HRR patterns exist between submaximal and maximal exercise paradigms among firefighters [6], it is possible that body composition may influence post-maximal exercise HRR responses in a different manner. The current study also did not incorporate heat stress or utilize personal protective equipment, which are inherent to the occupational tasks of firefighting and place additional cardiac strain and physiological burden on firefighters [5]. As such, future research should include larger sample sizes of both firefighter recruits and active-duty firefighters from a variety of fire departments, and utilize both submaximal and maximal exercise paradigms, under conditions with and without heat stress and personal protective equipment, to further characterize the influence of body composition on HRR responses within this at-risk population of interest.

## 5. Conclusions

The purpose of this study was to cross-sectionally examine the influence of body composition on the HRR response among firefighter recruits following a submaximal stepping task. In contrast to previous research, results of the current study indicate that BF, and not BMI or WC, was significantly correlated with clinical ΔHRR indices indicative of PSNS reactivation within this cohort sample of firefighter recruits. Thus, measures of overall BF percentage, and not surrogate measures of obesity and/or central adiposity, may influence the early phase of ANS recovery among firefighter recruits. Therefore, prioritizing the reduction in BF, with less emphasis on BMI or WC, may be more impactful in improving the early phase of ANS recovery among firefighter recruits.

## Figures and Tables

**Table 1:** Participant characteristics, mean (SD).

Variable	All (*n* = 57)	Men (*n* = 51)	Women (*n* = 6)
Age, yrs	30.7 (4.9)	30.7 (4.7)	30.8 (6.8)
Height, cm	179.9 (6.7)	180.8 (6.4)	172.2 (2.9)
Body Mass, kg	87.4 (11.0)	89.4 (9.6)	70.8 (7.3)
BMI, kg·m^−2^	27.0 (2.8)	27.4 (2.6)	23.9 (2.1)
WC, cm	86.3 (7.8)	87.8 (6.7)	73.8 (3.9)
BF, %	15.2 (4.1)	14.2 (2.7)	24.2 (3.0)

BMI, body mass index; WC, waist circumference; BF, body fat.

**Table 2:** Descriptive HRR data, mean (SD).

**HRR Responses, b·min^−1^**	**All (*n* = 57)**	**Men (*n* = 51)**	**Women (*n* = 6)**
HR_0_	149.7 (14.0)	149.3 (14.0)	153.2 (15.8)
HR_15_	139.0 (14.9)	138.5 (14.7)	142.8 (17.0)
HR_30_	126.1 (16.2)	126.1 (16.3)	126.0 (16.8)
HR_45_	116.1 (15.8)	116.2 (16.1)	115.5 (14.5)
HR_60_	109.3 (15.9)	109.8 (16.0)	105.5 (15.9)
HR_120_	95.5 (14.1)	95.9 (13.9)	91.5 (16.6)
HR_180_	90.9 (13.5)	91.4 (13.2)	87.0 (16.9)
**HRR Indices, b·min^−1^**	**All** (***n* = 57**)	**Men** (***n* = 51**)	**Women** (***n* = 6**)
ΔHRR_30_	23.6 (8.6)	23.2 (8.4)	27.2 (10.0)
ΔHRR_60_	40.4 (9.3)	39.5 (8.8)	47.7 (10.8)
ΔHRR_120_	54.2 (10.1)	53.4 (9.7)	61.7 (11.7)
ΔHRR_180_	58.8 (11.2)	57.9 (10.7)	66.2 (13.3)

HR_0_, post-exercise heart rate; HR_15_, 15-s post-exercise heart rate; HR_30_, 30-s post-exercise heart rate; HR_45_, 45-s post-exercise heart rate; HR_60_, 60-s post-exercise heart rate; HR_120_, 120-s post-exercise heart rate; HR_180_, 180-s post-exercise heart rate; HRR, heart rate recovery; ΔHRR_30_, 30-s heart rate recovery; ΔHRR_60_, 60-s heart rate recovery; ΔHRR_120_, 120-s heart rate recovery; ΔHRR_180_, 180-s heart rate recovery.

**Table 3:** Semi-partial correlations between body composition and HRR indices.

Variable	BMI, kg·m^−2^	WC, cm	BF, %
ΔHRR_30_, b·min^−1^	*r*_sp_ = −0.04, *p* = 0.395	*r*_sp_ = −0.12, *p* = 0.191	*r*_sp_ = −0.31, *p* = 0.010 *
ΔHRR_60_, b·min^−1^	*r*_sp_ = −0.08, *p* = 0.270	*r*_sp_ = −0.13, *p* = 0.174	*r*_sp_ = −0.27, *p* = 0.023 *
ΔHRR_120_, b·min^−1^	*r*_sp_ = −0.04, *p* = 0.385	*r*_sp_ = −0.06, *p* = 0.327	*r*_sp_ = −0.13, *p* = 0.175
ΔHRR_180_, b·min^−1^	*r*_sp_ = 0.03, *p* = 0.417	*r*_sp_ = −0.05, *p* = 0.354	*r*_sp_ = 0.01, *p* = 0.475

BMI, body mass index; WC, waist circumference; BF, body fat; HRR, heart rate recovery; ΔHRR_30_, 30-s heart rate recovery; ΔHRR_60_, 60-s heart rate recovery; ΔHRR_120_, 120-s heart rate recovery; ΔHRR_180_, 180-s heart rate recovery. * *p* < 0.05

## Data Availability

The data presented in this study are available on request from the corresponding author.
